# Supplementary material for: The path to re-evolve cooperation is constrained in Pseudomonas aeruginosa
Source: BMC Evol Biol. 2017 Sep 11;17:214. doi: 10.1186/s12862-017-1060-6 (PMC5594463; doi:10.1186/s12862-017-1060-6)
Supplement: Additional file 1: Figure S1. — Confirmed pyoverdine phenotypes in selected clones. Evolved clones with ancestral pyoverdine production levels were re-tested to confirm their phenotype. Figure depicts pyoverdine production measured in iron-limited media. Table S1. Clones selected for in-depth analysis and sequencing. Table containing additional information on clone identity, pyoverdine phenotype and pvdS sequencing for all 57 clones included in in-depth analysis. (PDF 695 kb) [file 12862_2017_1060_MOESM1_ESM.pdf]

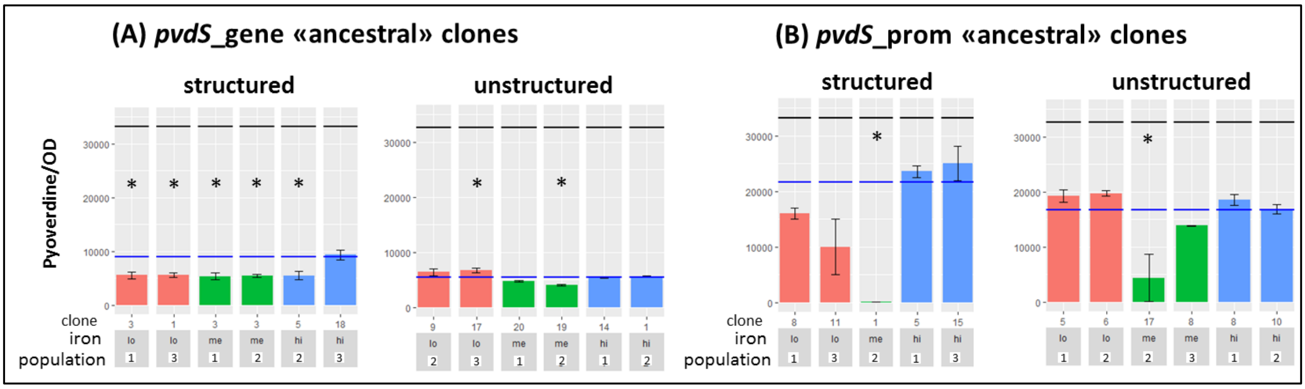

Fig. S1. Confirmed pyoverdine phenotypes in selected clones. Evolved clones with ancestral pyoverdine production levels were re-tested to confirm their phenotype. Pyoverdine production was measured in iron-limited media. **(a)** Clones evolved from the low-producer *pvdS*_gene. **(b)** Clones evolved from the low-producer *pvdS*_prom. Y axes show pyoverdine-specific fluorescence divided by growth (optical density at 600 nm) after 24 h of incubation. X axes show independent replicate populations the clones evolved in and iron availability during experimental evolution. Bars represent mean values of three replicates per evolved clone. Error bars denote standard error of the mean. The black line represents the average wildtype production level in the same assay, while the blue line denotes the average production level of the respective low-producing ancestor. Bars are coloured by iron availability during evolution: red = low iron; green = medium iron; blue = high iron. We used one-way ANOVAs with Tukey's post-hoc test for comparisons relative to the low-producing ancestor. Asterisks indicate a significant difference ($p<0.05$) from the ancestor.

Table S1. Clones selected for in-depth analysis and sequencing

Population[1]	clone #	pyoverdine phenotype[2]	*pvdS* mutation[3]	sequence length [bp][4]	*pvdS* status[5]	comment[6]
78_s_hi_2	5	ancestral	2722579 G>C	778	ancestral	
78_s_hi_3	18	ancestral	2722579 G>C; 2722596 A-->C	778	mutated	additional SNP in promoter region
78_s_lo_1	3	ancestral	2722579 G>C	778	ancestral	
78_s_lo_2	12	low	2722579 G>C	778	ancestral	
78_s_lo_3	1	ancestral	2722579 G>C	778	ancestral	
78_s_me_1	3	ancestral	2722579 G>C	778	ancestral	
78_s_me_2	3	ancestral	2722579 G>C	778	ancestral	
78_u_hi_1	14	ancestral	2722579 G>C	778	ancestral	
78_u_hi_1	8	high	2722579 G>C	778	ancestral	
78_u_hi_2	1	ancestral	2722579 G>C	778	ancestral	
78_u_hi_2	16	high	2722579 G>C	778	ancestral	
78_u_lo_1	1	low	2722579 G>C	778	ancestral	
78_u_lo_1	7	low	2722579 G>C	778	ancestral	
78_u_lo_1	12	low	2722579 G>C	778	ancestral	
78_u_lo_1	17	low	2722579 G>C	778	ancestral	
78_u_lo_1	19	low	2722579 G>C	778	ancestral	
78_u_lo_2	2	low	2722579 G>C	778	ancestral	
78_u_lo_2	5	low	N/A	N/A	N/A	sequencing failed
78_u_lo_2	8	low	2722579 G>C	778	ancestral	
78_u_lo_2	19	low	2722579 G>C	778	ancestral	
78_u_lo_2	9	ancestral	2722579 G>C	778	ancestral	
78_u_lo_3	14	low	2722579 G>C	778	ancestral	
78_u_lo_3	16	low	2722579 G>C	778	ancestral	
78_u_lo_3	17	ancestral	2722579 G>C	778	ancestral	
78_u_me_1	20	ancestral	2722579 G>C	778	ancestral	
78_u_me_1	16	high	2722579 G>C	778	ancestral	
78_u_me_2	19	ancestral	2722579 G>C	778	ancestral	
97_s_hi_1	2	low	2722079 G>T	778	ancestral	
97_s_hi_1	11	low	2722079 G>T	778	ancestral	
97_s_hi_1	12	low	2722079 G>T	778	ancestral	
97_s_hi_1	20	low	2722079 G>T	778	ancestral	
97_s_hi_1	5	ancestral	2722079 G>T	778	ancestral	
97_s_hi_2	11	high	2722079 G>T	778	ancestral	
97_s_hi_2	12	high	2722079 G>T	778	ancestral	
97_s_hi_2	5	low	2722079 G>T	778	ancestral	
97_s_hi_2	9	low	2722079 G>T	778	ancestral	
97_s_hi_2	14	low	2722079 G>T	778	ancestral	
97_s_hi_2	17	low	2722079 G>T	628	ancestral	incomplete sequence (-150 bp)
97_s_hi_2	20	low	2722079 G>T	778	ancestral	
97_s_hi_3	15	ancestral	2722079 G>T	778	ancestral	
97_s_lo_1	8	ancestral	2722079 G>T	778	ancestral	

97_s_lo_3	11	ancestral	2722079 G>T	778	ancestral	
97_s_me_2	7	low	2722079 G>T	778	ancestral	
97_s_me_2	1	ancestral	2722079 G>T	778	ancestral	
97_u_hi_1	8	ancestral	2722079 G>T	778	ancestral	
97_u_hi_2	10	ancestral	2722079 G>T	683	ancestral	incomplete sequence (-95 bp)
97_u_hi_3	2	low	2722079 G>T	778	ancestral	
97_u_hi_3	3	low	2722079 G>T	778	ancestral	
97_u_hi_3	8	low	2722079 G>T	642	ancestral	incomplete sequence (-136 bp)
97_u_hi_3	10	low	2722079 G>T	639	ancestral	incomplete sequence (-139 bp)
97_u_hi_3	12	low	2722079 G>T	639	ancestral	incomplete sequence (-136 bp)
97_u_hi_3	14	low	2722079 G>T	778	ancestral	
97_u_hi_3	19	low	2722079 G>T	778	ancestral	
97_u_lo_1	5	ancestral	2722079 G>T	628	ancestral	incomplete sequence (-150 bp)
97_u_lo_2	6	ancestral	2722079 G>T	778	ancestral	
97_u_me_2	17	ancestral	N/A	N/A	N/A	sequencing failed
97_u_me_3	8	ancestral	2722079 G>T	778	ancestral	

[1] 78 = *pvdS*_gene, 97 = *pvdS*_prom; s = structured; u= unstructured; lo = iron low; me = iron medium; hi = iron high

[2] pyoverdine phenotype compared to the low-producing ancestor in 1st screen (Fig. 3)

[3] genome location in PAO1 reference genome (*pseudomonas.com*) and respective change in nucleotide

[4] length of sequenced promoter region and *pvdS* gene; full sequence length = 778 bp (only *pvdS* gene: 564 bp)

[5] "ancestral": clone harbors only the mutation already present in the low-producing ancestor
"mutated": clone harbors mutation in addition to the one already present in the low-producing ancestor

[6] in case of incomplete sequencing, numbers in brackets indicate number of base pairs missing from the end of the *pvdS* gene
